# How to Enhance Government Trust and Social Cohesion: Evidence From China

**DOI:** 10.3389/fpsyg.2022.816019

**Published:** 2022-02-22

**Authors:** Juan Fan, Hanyi Zheng, Wenhui Liang

**Affiliations:** School of Journalism and Communication, Shanghai International Studies University, Shanghai, China

**Keywords:** impression management, government trust, social cohesion, education level, social psychology

## Abstract

Governments’ impression management behaviors are becoming increasingly common. Under this context, this study empirically analyzes the relationship between governments’ impression management behavior, citizens’ government trust and social cohesion by using questionnaire data based on social psychology theories. The conclusion shows that governments’ impression management behaviors positively affect citizens’ government trust and social cohesion. Government trust plays a mediating role between impression management behaviors and social cohesion, and citizens’ education levels positively moderate the relationship between governments’ impression management and citizens’ government trust, i.e., the higher the education level, the stronger the positive effect of governments’ impression management on citizens’ government trust. The findings of the study will provide significant references for the self-presentation of government information, citizens’ government trust and the improvement of social cohesion.

## Introduction

In a highly interactive online world with rapid information flows, the public is rather likely to be emotionally aroused; once the government lacks the corresponding impression management motives with poor responsiveness, misunderstandings may easily emerge among citizens, which then seriously erode the trust in local governments and the overall social cohesion. How to deliver effective self-presentation to enhance citizens’ trust in governments and social cohesion, and thus maintaining social security, has become a question to be re-examined by researchers and practitioners in the new age. Therefore, the impression management is getting increasing attention from politicians, political parties and government departments.

Impression management is regarded to be the result of the influence of information on managers’ behaviors (Merkl-Davies et al., 2011). Since the 1950s, the phenomenon of impression management began to draw the attention of social psychologists. Empirical social psychologists took the connection between people and their corresponding contexts as the entry point and applied this concept to a wide range of social phenomena to uncover the nature of human interaction (Liu, 2006). The development of research on individual impression management has laid the foundation for the research on organizational impression management, and the question that how organizations influence audiences’ perceptions through their behavioral and information moderation has become a new area of impression management research (Zhang et al., 2008). However, most of the existing studies have focused on enterprises, while fewer have put the topic in the context of governments; even for the few that do, they have only deemed impression management as a way of building the government image, while ignoring the mediating mechanisms and boundaries of its effects on citizens’ perceptions and behaviors (Meng et al., 2016; Pan, 2018). The lack of large sample data further makes empirical studies of impression management on citizens’ trust in government and social cohesion in the context of government behavior even more scarce, requiring further exploration.

Based on the background, this study constitutes an interdisciplinary exploration on impression management, government trust and social cohesion. By drawing on theoretical insights from social psychology, quantitative tests using the questionnaire, and structural equation modeling, it tries to analyze the relationship between governments’ impression management, government trust and social cohesion, revealing the implied mechanism, providing the theoretical guidance for practices. Based on the psychological perception level of the public, this study complements the narrow management and political science research by considering governments’ impression management behavior as influenced by social biases arising from others’ perceptions.

## Theoretical Background and Hypotheses

### Impression Management Theories

The concept of impression management was introduced by Goffman (1959), who argued that “impression management is like theater” and focused on the role of self-representation in daily life. Jones and Pittman (1982) extended impression management from the sociological realm to the psychological realm and suggested that self-presentation also involves one’s attempts to control others’ impressions of his/her personal characteristics. Social psychologists Leary and Kowalski (1990) suggested that impression management consists of two distinct processes, namely, impression motivation, which refers to an individual’s desire to control the perceptions and impressions that others form of him or her, and impression construction, i.e., “choosing the type of impression to be created” and putting it into practice. On this basis, Tetlock proposed a three-component model adding “impression monitoring”, indicating that individuals consciously seek to make a certain impression on others and keep a close eye on others’ impressions of themselves.

With the development of impression management theories, more and more scholars have applied these theories to the analysis at the organizational level, and took enterprises as the main objects of analysis to explore the drivers and impacts of the impression management behavior of enterprises (Jin, 2018). Some researchers have already proved the applicability of impression management at the macro-organizational level. However, there are seldom studies that have investigated the impression management behavior of governments as study objects, and even among the very few that do exist, they mainly adopted case studies to analyze the improvement of government image in cyberspace (Landtsheer et al., 2008; Geng, 2014). Allen and Caillouet proposed different impression management behaviors for different stakeholders, where the improvement of impression management behaviors are designed to improve images while protective impression management behaviors to reduce damage to images (Mohamed et al., 1999). This study focused on the impact of the improvement of impression management behaviors.

### Government Trust and Impression Management

As a kind of trust relationship, government trust is the psychological state and attitude that the public holds toward the government at the level of expectations and perceptions (Hetherington and Husser, 2012). What impression a government presents and how the impression is constructed influence the credibility and governing power of the government (Geng, 2014). A country’s foreign policy often affects the impression of the people of other countries (Hadaś-Dyduch, 2019; Minardi and Lestari, 2019). Citizen’s government trust can be enhanced through improving forms of information dissemination and impression management is exactly the strategic demonstration of information (Sallot, 2002). The impression management behavior of government enhances public trust in local government by influencing the public’s perception of governments, making them think that their governments fit well with current social expectations and social norms (Dowling and Pfeffer, 1975). Zhang and Ip (2019) found that the relationship between city government’s performance in pibulic service and residents’ trust in government is of great significance to public management of Chinese cities. Furthermore, evaluation of government performance interacts with perception of corruotion to negatively influence political trust (Wang, 2016). Therefore, the following hypothesis was proposed:

H1: Governments’ impression management positively affects the public trust in government.

### Government Trust and Social Cohesion

Cohesion the degree of mutual attraction, identification and support, which leads to emotional resonance or behavioral agreement based on common values, common goals, and common interests (Back, 1984; Li Y. N., 1998). No society can sustain and develop without public trust, support and commitment which are means that reduce the cost of political governance and effectively promote political solidarity and social integration. Political identification or trust as important factors influencing social cohesion (Li Y. S., 1998). Social cohesion is the degree of dependence, cooperation and unity among members of a society. It is particularly important for countries in transition (Jenson, 1998). The existence of groups which have grievancFes threatens social cohesion (Sapsford et al., 2019). The evidence suggests that governments that are not responsive to the expressed needs and wishes of their people do eventually fail (Acemoglu and Robinson, 2012). Huang directly regarded government trust as a dynamic indicator for indirectly interpreting changes in social cohesion. Some industrial management studies have confirmed the relationship between enterprise management and enterprise cohesion (Kovacova et al., 2020; Kovacova and Lewis, 2021). As a result, the following hypothesis was proposed:

H2: Trust in local government positively affects the social cohesion of the public.

### The Moderating Effect of Education Level

Studies have been conducted to analyze the factors influencing government trust, primarily from three aspects, namely, performance factors, cultural factors, and individual factors. Among them, individual factors mainly include individual characteristics and cognitive ability levels, specifically gender, age, education level, place of origin, and personality of individuals. Based on data from a large-scale questionnaire survey of Chinese urban citizens, Zhao employed linear regression and found that citizens with low education levels had higher government trust compared to those with high education levels (Zhao and Wang, 2019). Zhong and Chen (2002) suggested that better educated people were more rational and more skeptical of the information presented by governments and they are inclined to rationally analyze governments’ impression management behavior, thus their government trust tended to have smaller increases. Thus, the following hypothesis was proposed:

H3: Educational level negatively moderates the relationship between governments’ impression management behavior and the public trust in government, i.e., the higher the educational level, the weaker the above relationship is, and vice versa.

## Materials and Methods

### Research Approach

In this study, we used a questionnaire survey analysis approach. The official questionnaire was finalized and the formal research kicked off in November 2018. The questionnaires were distributed online to students enrolled in the Songjiang University Town in Shanghai, and 390 questionnaires were collected in total, of which 336 were valid.

### Variables Measurements

The questionnaire consisted of two parts. The first part contained questions for demographic data, including the gender, age, and education level. The second part contained questions for the main variables of the study, including governments’ impression management behavior, government trust, and social cohesion.

The independent variable was “governments’ impression management”, which was based on the scale developed by Goffman (1959) and Pan et al. (2017), and adjusts it according to government scenarios, forming “Government departments will convey care to the public” “The publicity issued by government departments to the society is vivid and cordial” and other 5-item scale measurements (α = 0.85); the dependent variable was “social cohesion”, which was based on the scale developed by Jenson (1998), Wang (2013) and Zhu (2007), forming “I think that members of society are willing to help each other” “I love our motherland very much” and other 5-item scale measurements (α = 0.74); the mediating variable was “government trust”, which was based on the scale developed by Gao (2014), forming “I think the government is willing to listen to the opinions of the people” “I think the government handles things fairly and appropriately” and other 5-item scale measurements (α = 0.90). All scales were evaluated on a 7-point Likert scale, ranging from 1 = “completely disagree” to 7 = “strongly agree”.

The moderating variable in this study was citizens’ education level, comprising four categories of the below-university level, bachelor’s level, master’s level, and doctoral level.

Moreover, factors such as citizens’ gender, age, and household income have been proved to affect citizens’ trust in government and social cohesion, and were thus included in the model as control variables in this study (Zhu, 2007; Wang, 2013; Wu et al., 2016).

## Results

### Descriptive Analyses

In order to understand the general characteristics of the samples and the overall performance of the main variables, the study first conducted a descriptive statistical analysis of the samples as well as the key variables. Based on the statistics, 52% (*n* = 187) of the samples studied were female, and 48% (*n* = 173) were male, representing a basically reasonable gender ratio in line with reality. Among them, the largest age group was the group of 16 to 27 years old, of whom the group of 19 years old took the majority, totaling 84, followed by the group of 18 years old, totaling 67, then the group of 20 years old, totaling 50, finally the group of 16 years old, with 1 person only. As to household income, the majority was concentrated in the range of 100,000–200,000 RMB, totaling 142 persons, followed by a total of 132 persons in the range of less than 100,000 RMB, 54 persons in the range of 200,000–300,000 RMB, 24 persons in the range of 300,000–400,000 RMB, 3 persons in the range of 400,000–500,000 RMB, and 6 persons in the range of more than 500,000 RMB. Table 1 shows the means, standard deviations, and Pearson correlation coefficients of all variables in this study.

**TABLE 1:** Means, standard deviations, and pearson correlation coefficients of all variables.

S.N.	Variable	Mean	S.D.	1	2	3	4	5	6	7
1	Governments’ impression management	6.39	0.63	1						
2	Trust in local governments	4.96	0.92	0.20**	1					
3	Social cohesion	6.13	0.75	0.44**	0.37**	1				
4	Education level	2.26	0.51	0.10	–0.02	0.06	1			
5	Gender	0.82	0.38	0.10	–0.06	0.13*	0.05	1		
6	Age	20.36	2.10	0.01	–0.05	0.001	0.76**	–0.004	1	
7	Household income	1.99	1.05	–0.10	−0.15**	−0.18**	−0.21**	0.03	−0.13*	1

“*” Correlation is significant at the 0.05 level (2-tailed). “**” Correlation is significant at the 0.01 level (2-tailed).

### Hypothesis Testing

Due to the complex structure of the model, and the mediating and moderating relationships between the constructs, the hypothesis testing in this study was performed using the PLS-SEM method. The R square of the dependent variable represented the predictability of the theoretical mode (Chin, 1998). The analysis revealed that the R^2^ of social cohesion was 17% and the R^2^ of government trust was 8%.

**FIGURE 1:**
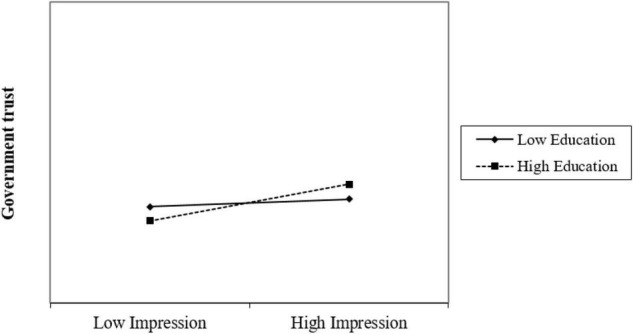
Moderating effect of education levels.

**FIGURE 2:**
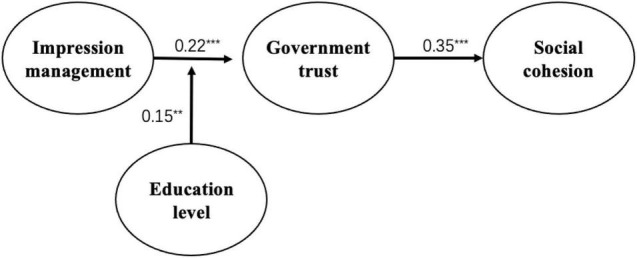
Model path coefficient diagram.

According to Figure 2, the results of data analysis showed that governments’ impression management positively affected the public trust in government (β = 0.22, *p* < 0.001), public trust in government positively affected their social cohesion (β = 0.35, *p* < 0.001), and citizens’ education levels positively moderated the relationship between governments’ impression management and government trust (β = 0.15, *p* < 0.05). Thus, H1 and H2 were verified, while H3 was not, with the moderating effect graph shown in Figure 1 below. According to Figure 1, the effect of governments’ impression management on citizens’ trust in government increased with citizens’ education. One possible explanation is that individuals with higher education levels enjoy more social capital and are more willing to participate in public affairs, which also reflects a higher level of trust in government among this group.

### Robustness Testing

Preacher and Hayes (2004) deemed the Bootstrap method as a more robust and effective way to test mediated effects with moderation. Therefore, this paper performed a robustness test on the data using the PROCESS program developed by Hayes. The “Bootstrap” analysis in this study was performed on 5000 replicate samples with a confidence interval of 95%. If the upper and lower limits of the confidence interval included zero, the effect tested was considered insignificant. The mediating effect was first tested, with the results of the data analysis shown in Table 2. It can be seen that the confidence intervals did not include 0, no matter with low, median or high government trust, indicating that the mediating effect of government trust between governments’ impression management and social cohesion was significant.

**TABLE 2:** Analysis of the mediating effect.

Mediating variable	Education level	Effect	S.D.	Lower limit	Upper limit
Government trust	Low	0.0368	0.0217	0.0012	0.0892
Government trust	Medium	0.0684	0.0220	0.0316	0.1202
Government trust	High	0.0999	0.0327	0.0461	0.1781

According to Table 3, the judgment indicator of the existence of moderating effect of education on the indirect relationship of governments’ impression management affecting social cohesion was 0.062 [confidence interval (0.003, 0.139)], with no 0 and with significance, thus confirming mediating effect with moderation. Therefore, the government trust of people with higher education is more likely to be affected by the behavior of government impression management; The political trust of people with low education level is not easy to be affected by the behavior of government impression management. This may be because people with low education level do not pay more attention to government behavior than those with high education level, and they do not understand the complex reasons behind government behavior.

**TABLE 3:** Test on the mediating effect with moderation.

Mediating variable	Effect	S.D.	Lower limit	Upper limit
Education level	0.062	0.033	0.003	0.139

## Discussion

Theoretically based on impression management, government trust and social cohesion, this study follows the logic of “management behavior-subjective cognition-behavioral response” and uses questionnaire data and PLS-SEM analysis to verify the relationship between government impression management behavior, citizens’ trust in government and social cohesion, and discusses the boundary conditions of the effect of government impression management on citizens’ trust in government. The boundary conditions of the role of impression management on citizens’ government trust were explored. The results of the large-sample statistical analysis showed that government impression management behavior positively influenced citizens’ government trust and social cohesion, government trust played a mediating role between impression management behavior and social cohesion, and citizens’ education level positively moderated the relationship between government impression management and citizens’ government trust, i.e., the higher the education level, the stronger the positive effect of government impression management on citizens’ government trust.

Compared with previous studies, this study has explored three new theoretical contributions. First, the study expands the perspective of social psychology research on government trust by exploring the influencing factors enhancing government trust based on the impression management theories. Second, the study has introduced the moderating variable of educational level to clarify the boundary of the role of impression management in the field of government trust research. Finally, the study has constructed a theoretical model of “Governments’ impression management behavior – Public trust in government – Social cohesion” by applying statistical analysis of large samples. In addition, this research also have significant practical implications. Impression management strategies can be one of the tools for local governments to communicate with the public. Especially at this special time when the COVID-19 poses great challenges to government operations globally and triggers widespread tensions between local governments and citizens, the use of appropriate self-presentation can make it easier for governments to obtain citizens’ understanding and increase their trust, thus enhancing social cohesion, reducing social panic and anxiety, and providing a solid cognitive foundation for smoothly surviving the pandemic. On top of that, the positive relationship between impression management strategies and government trust may vary with individuals’ education levels. Therefore, local governments should consider the educational characteristics of individuals and adopt different impression management behaviors for different groups of people.

This study does have certain limitations that can be further explored and bridged in future research. First, the applicability of the findings of this study still needs further verification. This is because the respondents in this study were mainly from the eastern coastal region of China, which has a higher degree of economic development, institutional construction, and public service development. Moreover, the main subject of this study is college students, which will limit the variable measurement of education level, without considering the “below-university level”. Second, limitations exist in the measurement of governments’ impression management behaviors in this study. This study mainly analyzed governments’ promotional impression management behaviors, not enough to present governments’ comprehensive impression management behaviors. Future research can further refine governments’ impression management behaviors based on the research scenarios. Third, the “continuity” of impression management strategies needs to be further confirmed. Government trust and social cohesion are influenced by a variety of complex factors, such as government responsiveness (Wang and Zheng, 2019), quality of government public services (Wu, 2017), government-public interaction (Mao and Zhu, 2019), and new online media (Sun and Chen, 2019), all of which have important effects on government trust. Although impression management penetrates all aspects of local government behaviors, the persistence of its role still needs to be verified by further research. Therefore, in future research, panel data are needed to verify the persistence of government impression management strategies.

## Data Availability Statement

The original contributions presented in the study are included in the article/supplementary material, further inquiries can be directed to the corresponding author.

## Author Contributions

JF: conceptualization, methodology, and writing-original draft preparation. HZ: investigation and writing-original draft preparation. WL: writing-reviewing and editing. All authors contributed to the article and approved the submitted version.

## Conflict of Interest

The authors declare that the research was conducted in the absence of any commercial or financial relationships that could be construed as a potential conflict of interest.

## Publisher’s Note

All claims expressed in this article are solely those of the authors and do not necessarily represent those of their affiliated organizations, or those of the publisher, the editors and the reviewers. Any product that may be evaluated in this article, or claim that may be made by its manufacturer, is not guaranteed or endorsed by the publisher.
